# Surgical Versus Rehabilitation-First Management Strategies After ACL Injury: Persisting Uncertainty over Long-Term Outcomes—A Systematic Search and Narrative Synthesis of Randomized Trial Cohorts

**DOI:** 10.3390/healthcare14091135

**Published:** 2026-04-23

**Authors:** Maciej Biały, Rafał Gnat

**Affiliations:** 1 Institute of Physiotherapy and Health Sciences, Academy of Physical Education, 40-065 Katowice, Poland; rafal.gnat@interia.pl; 2 ProRehab, 40-698 Katowice, Poland

**Keywords:** anterior cruciate ligament, ACL rupture, ACL reconstruction, non-operative treatment, rehabilitation, delayed reconstruction, randomized controlled trial, knee stability, osteoarthritis

## Abstract

**Background/Objectives**: The optimal management of anterior cruciate ligament (ACL) rupture remains debated, especially regarding long-term outcomes after early ACL reconstruction (ACLR) versus rehabilitation-first with optional delayed ACLR. The interpretation of randomized evidence is complicated by frequent treatment crossover. This review synthesized evidence from randomized controlled trial (RCT) cohorts comparing surgical versus rehabilitation-first management strategies across available follow-up durations. **Methods**: A structured review based on a systematic literature search and narrative synthesis was conducted, with study identification and reporting guided by PRISMA 2020. MEDLINE (via PubMed) and Google Scholar were searched in February 2026 for English-language human RCTs (2000–2026) comparing early ACLR plus rehabilitation with rehabilitation-first management allowing delayed ACLR for persistent instability. A linked-report PubMed search using the KANON trial registration number (ISRCTN84752559) was additionally performed to identify cohort-derived follow-up publications. Reports were grouped by underlying RCT cohort. Data were extracted on crossover, follow-up, and clinical outcomes. Risk of bias for primary RCT reports was assessed with Cochrane RoB 2. **Results**: Twenty-seven reports representing three RCT cohorts (KANON, COMPARE, ACL SNNAP) were included; six index reports were prioritized for synthesis. In acute ACL rupture (KANON, COMPARE), early ACLR did not show a consistent long-term superiority in patient-reported outcomes versus rehabilitation-first with optional delayed ACLR, although COMPARE reported a statistically significant 2-year subjective functional difference favoring early ACLR; early ACLR more consistently improved mechanical stability and reduced instability episodes. Crossover from rehabilitation to delayed ACLR was common. In non-acute ACL injury with persistent symptomatic instability (ACL SNNAP), surgery-first improved 18-month patient-reported outcomes. Meniscal procedure rates and osteoarthritis-related outcomes did not consistently favor early ACLR. **Conclusions**: In acute ACL rupture, rehabilitation-first with timely access to delayed ACLR appears to provide long-term patient-reported outcomes comparable to an early ACLR strategy in many patients, while early ACLR more consistently improves knee stability. In non-acute symptomatic ACL deficiency, a surgery-first strategy appears more effective in the mid-term. These randomized trials should be interpreted as comparisons of management strategies rather than of “pure” operative versus nonoperative treatment approaches.

## 1. Introduction

Anterior cruciate ligament (ACL) rupture is common in young, physically active populations and usually is associated with functional impairment as well as potential long-term consequences, including post-traumatic knee osteoarthritis (OA) and reduced quality of life [1,2]. Because many patients after ACL injury aim to return to pivoting or high-demand sports, initial treatment decision can have substantial implications for further knee joint function, activity/sports participation, and the risk of subsequent injury [1,3].

Healthcare pathways after ACL injury generally consist of two strategies: (1) early ACL reconstruction (ACLR) followed by rehabilitation, or (2) rehabilitation-first with the option of delayed ACLR for patients who develop symptomatic knee instability despite rehabilitation [1,4,5]. The arguments supporting early ACLR are based on restoration of mechanical stability and the potential reduction in recurrent “giving-way” episodes that may lead to secondary meniscal or chondral injury [6]. Conversely, a rehabilitation-first strategy may enable a substantial proportion of patients to achieve acceptable knee function, symptom control and return to activity/sports without surgery, avoiding surgery-related complications and additional costs [1,7].

It must be underlined that the evidence gathered from randomized controlled trials (RCTs) demonstrates that cross-over from nonoperative care to delayed ACLR is common, and that many patients allocated to rehabilitation do not need surgery during follow-up [4,5]. These features complicate interpretation of long-term treatment effectiveness, because cross-over can distort between-group contrasts in intention-to-treat analyses [4,5,8].

Irrespective of the treatment pathways, patients’ long-term outcomes are critical for evaluating comparative effectiveness of two different options, particularly in terms of OA development, meniscus injury and subsequent surgery risk, and achieved level of physical activity. At five years in the KANON randomized cohort (which provides the most extensive longitudinal dataset in the field including the longest follow-up and the largest number of secondary analyses), a strategy of early ACLR followed by structured rehabilitation did not yield superior outcomes compared with initial rehabilitation with optional delayed ACLR [9]. More recently, extended follow-up of this randomized cohort similarly reported no clear differences in patient-reported outcomes between groups [10]. Further data suggests that ACLR does not demonstrate a consistent long-term advantage for OA prevention compared with nonoperative management, although some analyses report differences in the objective measurement of knee laxity [11], function and/or subsequent meniscal procedures across strategies [2,12]. Nevertheless, uncertainty persists regarding long-term comparative outcomes, heterogeneity in rehabilitation protocols, variability in the subjective and objective outcomes used to define intervention success (e.g., patient-reported outcome measures, return-to-sport rates, knee laxity, objective functional outcomes, and subsequent injuries/surgeries), and inconsistent follow-up time points across studies [1,4,5,8,9,10,13]. Therefore, this review aimed to systematically identify and narratively synthesize evidence exclusively from RCTs comparing operative versus nonoperative management of ACL rupture across all reported follow-up durations.

## 2. Methods

This review was designed as a structured review based on a systematic literature search and narrative synthesis. Study identification, selection, and reporting were guided by PRISMA 2020. MEDLINE was searched via PubMed and Google Scholar was searched as a supplementary source; the search was last updated on 23 February 2026. Eligible records were restricted to English-language human randomized controlled trials published between 2000 and 2026 that compared two management strategies after ACL rupture: early ACLR plus structured rehabilitation versus a rehabilitation-first pathway with access to elective/delayed ACLR for persistent symptomatic knee instability. In PubMed, MeSH terms were used where applicable and were supplemented by title/abstract keywords for ACL rupture, operative treatment, nonoperative care, and staged timing/strategy concepts. Searches were restricted using PubMed filters for Humans, English, and Randomized Controlled Trial, and records focused only on postoperative rehabilitation after ACL reconstruction were excluded using title/abstract terms. Google Scholar was searched using two predefined queries, and because Google Scholar is not fully reproducible, the first 200 results per query sorted by relevance were screened and potentially eligible citations were exported and merged with PubMed records. To capture additional cohort-derived publications that may not be indexed as “Randomized Controlled Trial” (e.g., follow-up reports and secondary analyses from the same randomized cohort), an additional linked-report search was performed in PubMed using the KANON trial registration number (ISRCTN84752559) without the RCT publication-type filter. One reviewer conducted the search and initial screening of titles/abstracts and full texts, and any uncertainties regarding eligibility were resolved through discussion with the second author until consensus was reached. The full PubMed search strategy and predefined Google Scholar queries are provided in the Supplementary Materials. Records were excluded if they did not meet the predefined eligibility criteria, including randomized management-strategy design, relevant comparator pathways, human participants, English language, and the prespecified publication period; studies focused exclusively on postoperative rehabilitation after ACL reconstruction were also excluded. Eligible full texts were grouped into underlying randomized management-strategy trial cohorts. Multiple publications arising from the same randomized cohort were treated as separate “cohort-linked reports” but were counted as one independent “study” at the cohort level. The included evidence comprised three independent randomized cohorts: two in acute ACL rupture (KANON and COMPARE) and one in non-acute ACL injury with persistent symptomatic knee instability (ACL SNNAP). Because these populations represent clinically distinct scenarios, synthesis was organized at the cohort level and interpreted separately for acute versus non-acute presentations. Data were extracted on population characteristics, pathway details, cross-over/nonadherence, follow-up, and outcomes (patient-reported function as primary, with stability, meniscal/structural outcomes, OA-related outcomes, and adverse events as secondary outcomes). Risk of bias was assessed using Cochrane RoB 2 at the level of the three independent randomized trial cohorts, using the primary RCT report for each cohort (KANON, COMPARE, and ACL SNNAP). Cohort-linked follow-up reports and secondary analyses were not re-assessed as separate reports, because they arose from the same underlying randomized cohorts and did not represent independent randomized trials. These considerations were taken into account in the narrative synthesis, particularly in relation to the lack of blinding, crossover/nonadherence, and patient-reported outcomes. Due to heterogeneity and intrinsic cross-over in staged-care designs, results were synthesized narratively without meta-analysis.

## 3. Results

### 3.1. Study Selection

**Figure 1:**
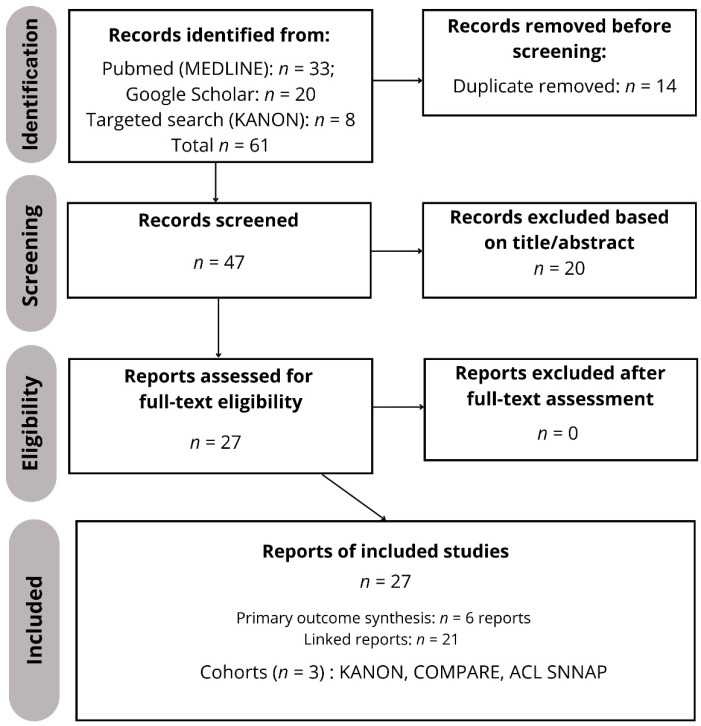
PRISMA 2020 flow diagram of the study selection process. From 61 identified records, 27 full-text reports were included. These 27 reports represent three unique randomized management-strategy trial cohorts (KANON, COMPARE, ACL SNNAP). In the PRISMA diagram, “reports” refers to individual publications, whereas “studies” refers to unique randomized cohorts (n = 3). For the narrative synthesis, we prioritized a predefined set of six index reports because they provide the primary functional outcomes at the main follow-up time points for each cohort (KANON 2, 5, and 11 years; COMPARE 24 months; ACL SNNAP 18 months) and one prespecified key secondary outcome report directly relevant to the review objectives (COMPARE meniscal procedures). The remaining cohort-linked reports were used to contextualize secondary domains without treating them as independent studies.

The PubMed search identified 33 records. Google Scholar was used as a supplementary source; two predefined queries returned 17,100 and 18,300 results, respectively. Because Google Scholar is not fully reproducible, the first 200 results per query (sorted by relevance) were screened and 20 records were exported (11 from query A and 9 from query B) and merged with PubMed records. Thus, 53 database-derived records entered the reference set; after removal of 14 duplicates, 39 records were screened by title/abstract and 20 were excluded, leaving 19 reports for full-text assessment. To capture cohort-derived publications that may not be indexed as randomized controlled trials, a linked-report search using the KANON trial registration number (ISRCTN84752559) was then performed in PubMed without the RCT publication-type filter, identifying eight additional KANON-linked reports. After adding these records, a total of 61 records had been identified overall; after deduplication, 47 records were screened, 20 were excluded, and 27 full-text reports were assessed. All 27 included reports could be linked to one of three underlying RCT cohorts (KANON, COMPARE, and ACL SNNAP); no eligible report fell outside these three cohorts. Multiple publications from the same cohort were treated as separate reports but counted as one study at the cohort level. Study selection is summarized in the PRISMA 2020 flow diagram (Figure 1).

### 3.2. Included Evidence

To maintain cohort-level methodological rigor, synthesis was organized at the cohort (study) level rather than the publication (report) level. For the primary functional outcomes, five index reports provided the main PROM endpoints at prespecified key time points: KANON (2-year primary report, 5-year follow-up, 11-year follow-up), COMPARE (24-month primary report), and ACL SNNAP (18-month primary report) [4,5,8,9,10]. One additional index report was prioritized because it addressed a core secondary objective of this review (meniscal procedures in COMPARE over 2 years) [13]. In total, 27 cohort-linked reports were included (KANON: 20; COMPARE: 4; ACL SNNAP: 3; listed in Supplementary Table S1). Importantly, Table 1 highlights the six reports used to anchor the main synthesis. The three included randomized cohorts differed in clinical profile, injury chronicity, management-strategy structure, primary outcome framework, follow-up duration, and crossover pathway. In particular, KANON and COMPARE enrolled patients with acute ACL rupture, whereas ACL SNNAP addressed non-acute ACL injury with persistent symptomatic instability. These distinctions are important for interpretation and applicability, and should be considered alongside the cohort-specific data summarized in Table 1 and described in the Results below. Risk of bias was evaluated for the three independent randomized cohorts, rather than separately for follow-up report or secondary analysis. Overall, RoB 2 judgments for the three randomized cohorts indicated some concerns, mainly related to lack of blinding, crossover/nonadherence, and reliance on patient-reported outcomes.

### 3.3. Trial Cohorts and Treatment Crossover

Because crossover from rehabilitation-first to delayed ACLR was common in the acute-injury cohorts (KANON, COMPARE), these trials should be interpreted as comparisons of management strategies (early ACLR vs. rehabilitation-first with optional delayed ACLR) rather than as contrasts between pure surgical and pure non-surgical treatment. KANON (2 years; acute ACL rupture): A total of 121 young, active adults were analyzed (early ACLR strategy, n = 62; rehabilitation with optional delayed ACLR, n = 59). In the optional-delayed group, 23/59 (37%) underwent delayed ACLR by 2 years (mean 11.6 months) [4]. At 5 years, follow-up data were available for 120/121 participants; 30/59 (51%) in the optional-delayed group had undergone delayed ACLR during the 5-year period [9]. At 11 years, follow-up included 53/62 participants in the early ACLR group and 54/59 in the optional-delayed group; among those followed, 28/54 (52%) in the optional-delayed group had undergone ACLR over the 11-year follow-up period [10]. COMPARE (2 years; acute ACL rupture): A total of 167 adults (18–65 years) were randomized to early ACLR (n = 85) or rehabilitation with elective delayed ACLR after ≥3 months if clinically indicated (n = 82). Two-year follow-up was completed by 98% of participants. In the rehabilitation group, 41/82 (50%) underwent ACLR during follow-up (mean 10.6 months after randomization) [5]. ACL SNNAP (18 months; non-acute symptomatic ACL deficiency): A total of 316 patients were randomized to a surgical management strategy (n = 156) or rehabilitation-based management (n = 160), with subsequent reconstruction permitted if instability persisted. In the rehabilitation group, 65/160 (41%) underwent ACLR within 18 months. In the surgical strategy group, 113/156 (72%) underwent reconstruction, whereas 43/156 (28%) did not undergo surgery [8].

### 3.4. Patient-Reported Outcome Measures

KANON (2 years; KOOS4 (Knee injury and Osteoarthritis Outcome Score) change): Mean KOOS4 change from baseline to 2 years was 39.2 (early ACLR) vs. 39.4 (rehabilitation with optional delayed ACLR), between-group difference 0.2 points (95% CI −6.5 to 6.8; *p* = 0.96) [4]. KANON (5 years; KOOS4 change): Mean KOOS4 change from baseline to 5 years was 42.9 (early ACLR) vs. 44.9 (optional delayed), between-group difference 2.0 points (95% CI −8.5 to 4.5; *p* = 0.54). No significant between-group differences were seen in KOOS4, KOOS subscales, SF-36, or Tegner activity scale at 5 years [9]. KANON (11 years; KOOS4): Mean KOOS4 improvement from baseline to 11 years was 46 points (early ACLR) vs. 45 points (optional delayed), between-group difference 1.6 points (95% CI −8.8 to 5.6; *p* = 0.67) [10]. COMPARE (2 years; IKDC): At 24 months, IKDC was 84.7 vs. 79.4 (difference 5.3, 95% CI 0.6 to 9.9; *p* = 0.026) favoring early ACLR; the time course differed, with IKDC favoring rehabilitation-first at 3 months (difference −9.3, 95% CI −14.6 to −4.0; *p* = 0.002) and favoring early ACLR at 9 months (difference 8.9, 95% CI 3.3 to 14.5). The authors noted that the clinical relevance of the 2-year difference was uncertain [5].

ACL SNNAP (18 months; KOOS4): Mean KOOS4 at 18 months was 73.0 (SD 18.3) in the surgical strategy group vs. 64.6 (SD 21.6) in rehabilitation; adjusted mean difference 7.9 (95% CI 2.5–13.2; *p* = 0.0053) favoring surgical management. All KOOS subscales also favored surgical management (pain difference 5.4; symptoms 6.8; ADL 8.1; sport/recreation 9.3; QoL 9.7) [8].

### 3.5. Knee Stability and Giving-Way

KANON (2 years): Objective stability favored early ACLR: KT1000 mean 6.6 mm vs. 8.3 mm (*p* = 0.001), normal Lachman 39/62 (65%) vs. 17/59 (29%) (*p* < 0.001), and normal pivot shift 45/62 (75%) vs. 27/59 (47%) (*p* = 0.003) [4]. KANON (5 years): Mechanical stability at rest at 5 years was significantly better with early ACLR strategy, normal Lachman was 45/59 (76%) after early ACLR, 18/30 (60%) after delayed ACLR, and 1/28 (4%) with rehabilitation alone (early ACLR vs. rehabilitation *p* < 0.001; delayed ACLR vs. rehabilitation *p* < 0.001; early vs. delayed *p* = 0.121), and normal pivot shift was 45/59 (76%), 18/30 (60%), and 5/28 (18%), respectively (early ACLR vs. rehabilitation *p* < 0.001; delayed ACLR vs. rehabilitation *p* = 0.002; early vs. delayed *p* = 0.121) [9]. KANON (11 years): Mechanical stability at 11 years continued to favor early ACLR (normal Lachman 33/49 [67%] vs. 17/51 [33%]; normal pivot shift 43/49 [88%] vs. 37/51 [73%]) [10]. COMPARE (2 years): Giving-way was reported in 2/81 (2.5%) in the early ACLR group vs. 12/80 (15.0%) in rehabilitation-first [5].

### 3.6. Meniscal Procedures

KANON (2 years): Total numbers of meniscal operations were 40 (early ACLR strategy) vs. 50 (optional delayed strategy), *p* = 0.20 [4]. KANON (5 years): Meniscus surgery occurred in 61/120 (51%) knees over 5 years: 29 in the early ACLR strategy group vs. 32 in the optional-delayed strategy group (*p* = 0.483), with no significant difference in time-to-event analysis (*p* = 0.774) [9]. COMPARE (during ACLR sessions): during ACL reconstruction sessions, 24 arthroscopic meniscus procedures were performed in the early ACLR group vs. 17 in the rehabilitation/optional-delayed group [5]. COMPARE (secondary analysis; 2-year meniscal procedures): baseline MRI showed a meniscal tear in 69/167 (41%) participants. During 2-year follow-up, 25/85 (29%) in early ACLR underwent a meniscal procedure vs. 17/82 (21%) in rehabilitation + optional delayed ACLR (RR 0.67, 95% CI 0.40–1.12; *p* = 0.12). Among those undergoing ACLR, meniscal procedures during reconstruction occurred in 23/82 (28%) after early ACLR and 13/41 (32%) after delayed ACLR; among patients with no ACLR (n = 41), 4/41 (10%) underwent isolated meniscal surgery during follow-up. Additional isolated meniscal procedures after ACLR occurred in 4/82 (5%) after early ACLR and 2/41 (5%) after delayed ACLR [13].

### 3.7. Osteoarthritis

KANON (2 years): No baseline radiographic OA was present. At 5 years, tibiofemoral radiographic OA developed in 13/113 (12%) and patellofemoral OA in 22/113 (19%), with no statistically significant differences between treatment strategies [9]. KANON (11 years): Radiographic OA of the injured knee was present in 44% of the cohort at 11 years, with no difference in the frequency of OA between strategies; however, mean summed incident radiographic OA feature scores were higher in the early ACLR group than in the optional-delayed group (2.4 vs. 1.4; mean difference 1.0, 95% CI 0.1 to 1.9) [10].

### 3.8. Adverse Events

KANON (2 years): Serious adverse events involving the index knee were 26 (early ACLR strategy) vs. 40 (optional delayed strategy), *p* = 0.06; within these, subjective/clinical instability events were 2 vs. 19, and meniscal signs/symptoms were 1 vs. 13 [4]. COMPARE (2 years): Serious adverse events included ACL re-rupture (four vs. two) and contralateral ACL rupture (three vs. one) in early ACLR vs. rehabilitation/optional delayed, respectively [5]. ACL SNNAP (18 months): Three graft failures were reported (two in the surgical group, one in rehabilitation). Newly acquired meniscal pathology occurred in one surgical and three rehabilitation participants. No ethics-reportable adverse events or serious adverse events were reported [8].

KANON (11 years): Late (5–11 years) knee injuries were self-reported at the 11-year follow-up, with recall acknowledged as a limitation [10].

## 4. Discussion

This systematically searched, narratively synthesized review of ACL injury management strategy indicates that, in acute ACL rupture, routine early ACLR does not improve long-term patient-reported outcomes compared with a rehabilitation-first approach that allows delayed reconstruction when needed. In KANON, improvements in KOOS4 were similar between strategies at 2, 5, and 11 years despite substantial crossover from rehabilitation-first to delayed surgery, suggesting that many patients can achieve acceptable symptoms and function gains without immediate reconstruction when followed closely and offered delayed ACLR for persistent instability [4,9,10]. In COMPARE, early ACLR resulted in a statistically significant but small advantage in IKDC at 24 months, with a time course that initially favored rehabilitation-first and later favored early surgery, and with half of participants assigned to rehabilitation avoiding reconstruction within 2 years. [5] By contrast, in the non-acute symptomatic population studied in ACL SNNAP, a surgery-first strategy provided superior KOOS4 at 18 months, supporting the concept that chronicity and symptomatic knee instability substantially modify comparative effectiveness [8]. Across cohorts, mechanical stability outcomes tended to favor early reconstruction, whereas differences in long-term joint structure (radiographic OA) remained uncertain, reinforcing the need for individualized, symptom-guided decision-making rather than a single universal strategy [4,9,10].

### 4.1. Acute Versus Non-Acute ACL Injury

The divergence between the acute-injury trials (KANON and COMPARE) and ACL SNNAP is likely explained by differences in patient baseline clinical phenotype and timing. Acute cohorts include a heterogeneous mix of patients, some of whom can compensate for ACL deficiency with structured rehabilitation and activity modification, while others develop recurrent giving-way that prompts conversion to delayed reconstruction; this is reflected in the substantial crossover rates observed in both KANON and COMPARE [4,5,9,10]. In contrast, ACL SNNAP enrolled patients with non-acute ACL injury and persistent symptomatic knee instability. These participants are less likely to succeed with rehabilitation alone and more likely to benefit from surgical stabilization, which may partly explain the clearer advantage of ACLR first on KOOS4 at 18 months [8]. Therefore, the trials should not be interpreted as conflicting evidence, but rather as complementary findings across different clinical scenarios: an initial rehabilitation-first pathway appears reasonable for many acute ACL ruptures provided that instability is monitored and timely delayed ACLR is available, whereas primary reconstruction is more strongly supported when symptomatic instability is already established in a non-acute population [5,8].

### 4.2. Improved Knee Stability Does Not Necessarily Translate into Better Functional Outcomes

Across cohorts, patient-reported outcome measures and knee mechanical stability did not move in parallel. In KANON, KOOS4 improvements were essentially identical between early ACLR and rehabilitation-first strategies at 2, 5, and 11 years, despite consistently better knee mechanical stability in the early ACLR group, indicating that superior laxity/pivot-shift findings did not translate into superior long-term PROMs in this cohort [4,9,10]. In COMPARE, early ACLR resulted in a statistically significant but small IKDC advantage at 24 months, while giving-way complaints were more frequent in the rehabilitation-first arm, illustrating that perceived function and symptom trajectories are not determined by stability metrics alone [5]. In ACL SNNAP, conducted in a non-acute symptomatic population, KOOS4 favored surgery-first at 18 months, which is consistent with the clinical expectation that established symptomatic instability is more likely to benefit from surgical stabilization [8]. Complementary long-term observational evidence in high-level athletes supports these findings: at 10 years in a matched-pair design, operative treatment produced better clinical stability but no differences in activity level, or subjective and objective functional outcomes compared with conservative treatment [14], and at 20 years in a pair-matched cohort (treatment allocated according to response after 3 months of rehabilitation), knee stability was clearly better in the operative group, yet subjective function (including IKDC subjective and KOOS) and osteoarthritis prevalence did not differ between operative and nonoperative management [11]. A separate 10-year randomized trial in non-athletes similarly reported comparable KOOS and IKDC between ACLR and conservative care at final follow-up, despite more favorable clinical stability after reconstruction [15]. Overall, these patterns align with contemporary evidence syntheses indicating that ACL reconstruction tends to improve objective stability, whereas superiority in patient-reported outcomes over non-surgical management is limited or inconsistent, particularly when analyses account for rehabilitation quality and staged crossover pathways [12,16,17]. Because the 10- and 20-year athlete studies are observational (matched-pair) with treatment allocation linked to early response and historical surgical techniques, their findings should be interpreted as supportive context rather than causal proof, but they reinforce the central point that better mechanical knee stability does not automatically translate into better PROMs [11,14].

### 4.3. Meniscus and Secondary Injury Risk

Meniscal preservation is central to long-term joint health after ACL rupture, and a common rationale for early ACLR is the prevention of secondary meniscal damage triggered by recurrent giving-way episodes [4,5]. In the KANON, meniscal outcomes did not clearly favor early ACLR: the total number of meniscal operations over 2 years was similar between strategies (40 vs. 50), and at 5 years meniscus surgery rates and time-to-event analyses remained comparable (29 vs. 32) despite superior mechanical stability in the early ACLR group [4,9]. In COMPARE, a linked secondary analysis likewise found no statistically significant increase in meniscal procedures over 2 years with rehabilitation-first and optional delayed ACLR compared with early ACLR (29% vs. 21%), even though baseline MRI showed meniscal tears in 41% of participants and 50% of the rehabilitation-first group crossed over to ACLR during follow-up [5,13]. At the same time, the higher frequency of giving-way in the rehabilitation-first arm in COMPARE provides a clinical pathway for secondary injury in a subset of patients, emphasizing the importance of early identification of persistent symptomatic instability and timely referral to surgery when needed [5]. In SNNAP, newly acquired meniscal pathology events during follow-up were uncommon, with only 1 event in the surgical group versus 3 in the rehabilitation group [18]. Beyond these RCTs, longer-term observational evidence is mixed but informative regarding secondary meniscal tears: a large cohort study reported lower risks of secondary meniscal tears (and downstream symptomatic arthritis/TKA outcomes) after ACL reconstruction compared with nonoperative management, with early ACLR appearing more protective than delayed ACLR [19]. A subsequent long-term cohort analysis reported that secondary meniscal tears were most common among patients managed nonoperatively or with delayed ACLR and were frequently complex medial tears associated with high rates of partial meniscectomy [20]. However, matched-pair studies in high-level athletes did not demonstrate clear differences in meniscal lesions/meniscectomy at 10–20 years despite better stability after operative treatment [11,14]. Taken together, evidence from randomized settings suggests that a rehabilitation-first strategy with optional delayed ACLR does not necessarily increase meniscal surgery within 2–5 years when follow-up is close and delayed surgery is accessible, while observational cohorts suggest that prolonged instability or delayed reconstruction may increase secondary meniscal tear risk in some settings [9,13]; therefore, extended follow-up of randomized cohorts is needed to capture delayed meniscal pathology and meniscal procedures that may not become apparent within short-to-mid-term time horizons.

### 4.4. Osteoarthritis

Post-traumatic OA remains a major concern after ACL rupture, yet the randomized evidence reviewed here does not support ACLR as a reliable strategy to prevent long-term degenerative change [9,10]. In KANON, radiographic OA was absent at baseline, but tibiofemoral OA and patellofemoral OA were detectable by 5 years (12% and 19%, respectively) without statistically significant differences between early ACLR and rehabilitation-first strategies [9]. At 11 years, radiographic OA of the injured knee was present in 44% of the cohort and the frequency of OA did not differ between strategies, although the mean summed incident radiographic OA feature score was higher after early ACLR than after rehabilitation-first with optional delayed ACLR (2.4 vs. 1.4; mean difference 1.0, 95% CI 0.1–1.9) [10]. Linked structural analyses from the same randomized cohort provide additional nuance, suggesting that structural cartilage changes can occur irrespective of strategy assignment: patellofemoral cartilage thickness loss over 5 years was greater in the early ACLR strategy than in the optional delayed strategy in a secondary analysis, indicating that early surgery did not prevent early patellofemoral structural deterioration [21], while another 5-year MRI analysis examined tibiofemoral cartilage thickness change as a structural outcome within the same cohort [22]. A post hoc KANON analysis further suggested that varus alignment after ACL rupture was associated with subsequent medial tibiofemoral OA, reinforcing that OA risk is influenced by loading/alignment factors beyond the initial treatment strategy [23]. External long-term evidence is broadly consistent with the lack of a clear OA preventive effect: a small, randomized comparison with 10-year follow-up reported no difference in radiographic OA between surgical and non-surgical management despite better stability after ACLR [24], and other long-term comparative cohorts have similarly questioned whether reconstruction alters the long-term OA trajectory after ACL injury [25]. Contemporary syntheses also align with this interpretation, generally concluding that reconstruction improves stability but does not confer a consistent advantage for OA prevention compared with nonoperative treatment [17,26].

### 4.5. Clinical Implications

Presented findings support a pragmatic, individualized approach rather than a single universal strategy [4,5,8]. For many patients with acute ACL rupture, a rehabilitation-first pathway with close monitoring and timely access to delayed ACLR can achieve comparable long-term PROMs while avoiding immediate surgery in a substantial proportion [5,9,10]. Early ACLR may be more appropriate when knee instability is frequent or unacceptable, when pivoting/cutting sport demands are high, or when functional stability cannot be achieved despite structured rehabilitation [5,9]. In non-acute ACL deficiency with persistent symptomatic instability, the ACL SNNAP results support offering reconstruction without mandatory delay, as the average functional benefit favored ACLR [8]. Further research is needed to identify which patient subgroups are most likely to benefit from ACLR versus rehabilitation-first management and to clarify the long-term meniscal and osteoarthritis-related outcomes.

### 4.6. Methodological Considerations and Limitations

Interpretation of the included trials and of this review requires attention to design features inherent to management-strategy RCTs after ACL rupture and to the limitations of the evidence base [4,5,8]. Blinding is not feasible for surgery versus rehabilitation and primary outcomes are patient-reported, which may introduce expectation and performance effects. Crossover and nonadherence are not protocol deviations but integral to staged care pathways; therefore, intention-to-treat estimates reflect the effect of assignment to a strategy rather than the pure biological effect of “received ACLR” and between-group contrasts may be diluted when many participants cross over. Heterogeneity across cohorts (acute versus non-acute populations, different primary PROM instruments, and different follow-up horizons) limits quantitative pooling and supports a narrative synthesis focused on clinical context and time course [5,8,9,10]. Strengths of this review include the focus on randomized strategy cohorts with long-term follow-up, including KANON data extending to 11 years, and a structured mapping of cohort-linked publications to capture follow-ups and secondary analyses [9,10]. Limitations include restricted database coverage (MEDLINE with supplementary Google Scholar), the non-reproducibility of Google Scholar search results, and the small number of eligible strategy RCT cohorts, which limits generalizability across settings and rehabilitation protocols. The search did not include a European database such as Embase, Scopus, or the Cochrane Library.

## 5. Conclusions

In acute ACL rupture, current randomized evidence does not show clear or consistent long-term superiority of early ACLR over a rehabilitation-first approach in patient-reported outcomes. Early ACLR more consistently improves knee stability and reduces episodes of instability. In non-acute ACL injury with persistent symptomatic instability, a surgery-first strategy appears to provide superior mid-term functional outcomes. Across both clinical scenarios, evidence does not demonstrate a substantial advantage of either strategy for meniscal preservation or osteoarthritis prevention.

## Figures and Tables

**Table 1:** Key outcomes from randomized management-strategy trials comparing early ACL reconstruction versus rehabilitation-first with optional delayed reconstruction.

Trial (Report)	Population	Group (n)	Follow-Up	Crossover	PROMs	Stability/Giving-way	Meniscus Outcomes	OA
KANON 2010 [4]	Acute ACL rupture; young active adults	Early ACLR + rehab (62) vs. rehab + optional delayed ACLR (59)	2 years	Delayed ACLR: 23/59 (37%)	KOOS4 change: 39.2 vs. 39.4; Between-group diff 0.2 (95% CI −6.5 to 6.8, *p* = 0.96)	KT1000: 6.6 vs. 8.3 mm (*p* = 0.001); Normal Lachman: 65% vs. 29% (*p* < 0.001); Normal pivot shift: 75% vs. 47% (*p* = 0.003)	Total meniscal operations: 40 vs. 50, *p* = 0.20	NR
KANON 2013 [9]	Acute ACL rupture; young active adults	Early ACLR + rehab (62) vs. rehab + optional delayed ACLR (59)/follow-up: 61/62 vs. 59/59]	5 years	Delayed ACLR: 30/59 (51%)	KOOS4 change: 42.9 vs. 44.9; Between-group diff 2.0 (95% CI −8.5 to 4.5, *p* = 0.54)	Mechanical stability favored early ACLR; Lachman/pivot shift (*p* < 0.001)	Meniscus surgery: 29 vs. 32, *p* = 0.483	No between-group difference; Overall TF OA 13/113 (12%), PF OA 22/113 (19%)
KANON 2023 [10]	Acute ACL rupture; young active adults	Early ACLR + rehab (62) vs. rehab + optional delayed ACLR (59)/follow-up: 53/62 vs. 54/59	11 years	Delayed ACLR: 28/54 (52%)	KOOS4 improvement (baseline→11 y): 46 vs. 45; Between-group diff 1.6 (95% CI −8.8 to 5.6, *p* = 0.67)	Normal Lachman: 33/49 (67%) vs. 17/51 (33%); Normal pivot shift: 43/49 (88%) vs. 37/51 (73%)	NR	Radiographic OA (index knee): 44% overall; no between-group difference; Mean summed incident OA feature score at 11 y: 2.4 vs. 1.4 (mean diff 1.0; 95% CI 0.1–1.9)
COMPARE 2021 [5]	Acute ACL rupture; adults 18–65 years	Early ACLR + rehab (85) vs. rehab + optional delayed ACLR (82)	2 years	Delayed ACLR 41/82 (50%)	IKDC at 24 months: 84.7 vs. 79.4; Between-group diff. 5.3 (95% CI 0.6 to 9.9, *p* = 0.026)	Giving-way at 2 years: 2/81 (2.5%) vs. 12/80 (15.0%)	COMPARE 2023	NR
COMPARE 2023 [13]	Acute ACL rupture; adults 18–65 years	Early ACLR + rehab (85) vs. rehab + optional delayed ACLR (82)	2 years	As per COMPARE 2021	NR (meniscus-focused report)	NR	Meniscal procedure/patient: 25/85 (29%) vs. 17/82 (21%); RR 0.67 (95% CI 0.40–1.12), *p* = 0.12; Baseline MRI meniscal tear 69/167 (41%)	NR
ACL SNNAP 2022 [8]	Non-acute ACL injury with persistent symptomatic knee instability	ACLR + rehab (156) vs. rehab-first (160)	18 months	ACLR received: 113/156 (72%); No ACLR surgery arm: 43/156 (28%); Rehab→surgery: 65/160 (41%)	KOOS4 at 18 months: 73.0 (SD 18.3) vs. 64.6 (SD 21.6); Between-group diff 7.9 (95% CI 2.5–13.2, *p* = 0.0053)	NR	New meniscal pathology: 1 vs. 3	NR

Table summarizes the six reports selected for the primary outcome synthesis. These reports were chosen based on their representation of key clinical follow-up milestones (ranging from 18 months to 11 years) and their focus on the primary research question. While the review identified 27 reports in total, the remaining 21 publications served as supplementary evidence and are detailed in the Supplementary Materials. Abbreviations: ACL, anterior cruciate ligament; ACLR, ACL reconstruction; IKDC, International Knee Documentation Committee score; KOOS4, mean of four Knee injury and Osteoarthritis Outcome Score subscales; NR, not reported; OA, osteoarthritis; PF, patellofemoral; PROMs, patient-reported outcome measures; TF, tibiofemoral.

## Data Availability

No new data were created or analyzed in this study. Data sharing is not applicable to this article.

## Supplementary Materials

The following supporting information can be downloaded at: https://www.mdpi.com/article/10.3390/healthcare14091135/s1, Methods S1. Search Strategy and Study Identification. Table S1. Publications linked to included RCT cohorts (primary reports, follow-ups, secondary analyses, and economic evaluations). Refs. [27,28,29,30,31,32,33,34,35,36,37,38,39,40,41,42] were cited in Supplementary Materials.
