# Supplementary material for: Surgical Versus Rehabilitation-First Management Strategies After ACL Injury: Persisting Uncertainty over Long-Term Outcomes—A Systematic Search and Narrative Synthesis of Randomized Trial Cohorts
Source: Healthcare (Basel). 2026 Apr 23;14(9):1135. doi: 10.3390/healthcare14091135 (PMC13163805; doi:10.3390/healthcare14091135)
Supplement: Supplementary file 1 [file healthcare-14-01135-s001.zip › healthcare-4231759-supplementary.pdf]

Supplementary Methods S1. Search Strategy and Study Identification

Search date

The literature search was last updated on 23 February 2026.

Information sources

MEDLINE was searched via PubMed, and Google Scholar was used as a supplementary source. In addition, a linked-report search was performed in PubMed using the KANON trial registration number (ISRCTN84752559) without restricting by publication type, in order to identify cohort-derived follow-up publications and secondary analyses that may not have been indexed as randomized controlled trials.

PubMed (MEDLINE) search strategy

In PubMed, MeSH terms and title/abstract keywords were combined for the following concepts:
(1) ACL rupture (e.g., “anterior cruciate ligament”/ACL with rupture/tear/injury),
(2) operative treatment (reconstruction/surgery/ACLR),
(3) non-operative care (rehabilitation/exercise therapy/nonoperative/conservative), and
(4) staged timing/strategy terms (early/delayed/elective/optional/“rehabilitation first”/strategy).

Search results were restricted using PubMed filters for Humans, English, and Randomized Controlled Trial, with publication years limited to 2000–2026.

The final PubMed search strategy was as follows:

((("anterior cruciate ligament"[MeSH Terms] OR "anterior cruciate ligament"[Title/Abstract] OR "ACL"[Title/Abstract]) AND ("rupture"[Title/Abstract] OR "tear"[Title/Abstract] OR "injury"[Title/Abstract]) AND ("reconstruction"[Title/Abstract] OR "surgery"[Title/Abstract] OR "ACLR"[Title/Abstract]) AND ("rehabilitation"[Title/Abstract] OR "exercise therapy"[Title/Abstract] OR "nonoperative"[Title/Abstract] OR "conservative"[Title/Abstract]) AND ("early"[Title/Abstract] OR "delayed"[Title/Abstract] OR "elective"[Title/Abstract] OR "optional"[Title/Abstract] OR "strategy"[Title/Abstract] OR "rehabilitation first"[Title/Abstract])) NOT ("postoperative"[Title/Abstract] OR "post-operative"[Title/Abstract] OR "after reconstruction"[Title/Abstract] OR "after ACL reconstruction"[Title/Abstract])) AND ((randomizedcontrolledtrial[Filter]) AND (humans[Filter]) AND (english[Filter]) AND (2000:2026[pdat]))

This search yielded 33 records.

Google Scholar supplementary search

Google Scholar was searched using two predefined queries:

Query A:
“anterior cruciate ligament” trial rehabilitation early reconstruction delayed

Query B:
ACL randomized trial rehabilitation versus reconstruction

These queries returned 17,100 and 18,300 results, respectively. Because Google Scholar results are not fully reproducible, the first 200 results per query, sorted by relevance, were screened. Potentially eligible citations were exported and merged with the PubMed records. In total, 20 records were exported from Google Scholar (11 from Query A and 9 from Query B).

Linked-report search

To capture additional publications arising from the same randomized cohort but not necessarily indexed as randomized controlled trials, an additional PubMed linked-report search was performed using the KANON trial registration number (ISRCTN84752559) without the RCT publication-type filter. This search identified 8 additional KANON-linked reports.

Linked cohort-derived publications included primary trial reports, follow-up reports, prespecified or exploratory secondary analyses (e.g., meniscal and structural outcomes), and economic evaluations.

Record management and selection

All citations identified from PubMed and Google Scholar were merged in a reference manager and deduplicated. After initial deduplication, 14 duplicates were removed. A total of 39 records then underwent title/abstract screening, of which 20 were excluded, leaving 19 reports for full-text assessment.

After inclusion of the linked-report search results, a total of 61 records had been identified overall. Following deduplication, 47 records were screened and 27 full-text reports were assessed and retained for inclusion.

All 27 included reports were linked to one of three underlying randomized management-strategy trial cohorts: KANON, COMPARE, and ACL SNNAP.

Reviewer workflow

One reviewer conducted the literature search and the initial screening of titles/abstracts and full texts. Any uncertainties regarding eligibility were resolved through discussion with the second author until consensus was reached.

Study identification framework

The study selection process is summarized in the PRISMA 2020 flow diagram (Figure 1). In the review, the term "reports" refers to individual publications, whereas "studies" refers to unique randomized cohorts.

Supplementary Table S1. Publications linked to included RCT cohorts (primary reports, follow-ups, secondary analyses, and economic evaluations)

RCT cohort	Report type	Outcome domain	Reference (short)	PMID	DOI
ACL SNNAP	Primary RCT report	KOOS4; satisfaction; events	Beard DJ 2022. Rehabilitation versus surgical reconstruction for non-acute anterior cruciate ligament injury (ACL SNNAP): a pragmatic randomised controlled trial. Lancet	35988569	10.1016/S0140-6736(22)01424-6
ACL SNNAP	Full trial report (HTA)	Methods + outcomes + sensitivity analyses	Beard DJ 2024. Comparison of surgical or non-surgical management for non-acute anterior cruciate ligament injury: the ACL SNNAP RCT. Health Technol Assess	38940695	10.3310/VDKB6009
ACL SNNAP	Economic evaluation	Cost-effectiveness	Leal J 2024. Cost-effectiveness analysis of a pragmatic randomized trial evaluating surgical reconstruction versus rehabilitation in patients with long-standing anterior cruciate ligament injury. Bone Joint J	38160685	10.1302/0301-620X.106B1.BJJ-2023-0175.R1
COMPARE	Primary RCT report	IKDC; giving-way; injuries	Reijman M 2021. Early surgical reconstruction versus rehabilitation with elective delayed reconstruction for patients with anterior cruciate ligament rupture: COMPARE randomised controlled trial. BMJ	33687926	10.1136/bmj.n375
COMPARE	Economic evaluation	Cost-effectiveness	Eggerding V 2022. ACL reconstruction for all is not cost-effective after acute ACL rupture. Br J Sports Med	33737313	10.1136/bjsports-2020-102564
COMPARE	Secondary analysis	Predictors of nonoperative failure	van der Graaff SJA 2022. Why, When, and in Which Patients Nonoperative Treatment of Anterior Cruciate Ligament Injury Fails: An Exploratory Analysis of the COMPARE Trial. Am J Sports Med	35048733	10.1177/03635465211068532
COMPARE	Secondary analysis	Meniscal procedures (2-year analysis)	van der Graaff SJA 2023. Meniscal procedures are not increased with delayed ACL reconstruction and rehabilitation: results from a randomised controlled trial. Br J Sports Med	36137731	10.1136/bjsports-2021-105235
KANON	Secondary analysis	Muscle strength / function / performance	Ageberg E 2008. Muscle strength and functional performance in patients with anterior cruciate ligament injury treated with training and surgical reconstruction or training only: a two to five-year followup. Arthritis Rheum	19035430	10.1002/art.24066
KANON	Qualitative study	Patient decision-making / preferences	Thorstensson CA 2009. Choosing surgery: patients' preferences within a trial of treatments for anterior cruciate ligament injury. A qualitative study	19664258	10.1186/1471-2474-10-100
KANON	Primary RCT report	PROMs; stability; meniscus; adverse events	Frobell RB 2010. A randomized trial of treatment for acute anterior cruciate ligament tears. N Engl J Med	20660401	10.1056/NEJMoa0907797
KANON	Secondary analysis	Physical performance	Ericsson YB 2013. Lower extremity performance following ACL rehabilitation in the KANON-trial: impact of reconstruction and predictive value at 2 and 5 years. Br J Sports Med	24029859	10.1136/bjsports-2013-092642
KANON	Follow-up report	PROMs; radiographic OA; meniscus	Frobell RB 2013. Treatment for acute anterior cruciate ligament tear: five year outcome of randomised trial. BMJ	23349407	10.1136/bmj.f232
KANON	Secondary	Bone shape /	Hunter DJ 2014. The effect of anterior	248676	10.1016/j.joca.2014.05.

	analysis	curvature	cruciate ligament injury on bone curvature: exploratory analysis in the KANON trial. Osteoarthritis Cartilage	33	014
KANON	Secondary analysis	Cartilage thickness changes (5-year follow-up)	Eckstein F 2015. Five-year followup of knee joint cartilage thickness changes after acute rupture of the anterior cruciate ligament. Arthritis Rheumatol	25252019	10.1002/art.38881
KANON	Brief report	Short report linked to KANON follow-up	Frobell RB 2015. Treatment for acute anterior cruciate ligament tear: five year outcome of randomised trial. Br J Sports Med	25926596	10.1136/bjsports-2014-f232rep
KANON	Secondary analysis	Muscle function vs future PROMs	Flosadottir V 2016. Muscle function is associated with future patient-reported outcomes in young adults with ACL injury. BMJ Open Sport Exerc Med	27900196	10.1136/bmjsem-2016-000154
KANON	Economic evaluation	Cost-effectiveness	Kiadaliri AA 2016. No economic benefit of early knee reconstruction over optional delayed reconstruction for ACL tears: registry enriched randomised controlled trial data. Br J Sports Med	26935859	10.1136/bjsports-2015-095308
KANON	Secondary analysis	Prognostic factors for outcome	Filbay SR 2017. Delaying ACL reconstruction and treating with exercise therapy alone may alter prognostic factors for 5-year outcome: an exploratory analysis of the KANON trial. Br J Sports Med	28515057	10.1136/bjsports-2016-097124
KANON	Secondary analysis	Synovial fluid cytokines (biomarkers)	Larsson S 2017. Surgical reconstruction of ruptured anterior cruciate ligament prolongs trauma-induced increase of inflammatory cytokines in synovial fluid: an exploratory analysis in the KANON trial. Osteoarthritis Cartilage	28522220	10.1016/j.joca.2017.05.009
KANON	Secondary analysis	Self-efficacy / performance	Flosadottir V 2018. Impact of treatment strategy and physical performance on future knee-related self-efficacy in individuals with ACL injury. BMC Musculoskelet Disord	29433481	10.1186/s12891-018-1973-2
KANON	Secondary analysis	Patellofemoral cartilage thickness loss	Culvenor AG 2019. Loss of patellofemoral cartilage thickness over 5 years following ACL injury depends on the initial treatment strategy: results from the KANON trial. Br J Sports Med	30737199	10.1136/bjsports-2018-100167
KANON	Secondary analysis	Perceived treatment success / outcome framing	Roos EM 2019. It is good to feel better, but better to feel good: whether a patient finds treatment 'successful' or not depends on the questions researchers ask. Br J Sports Med	31072841	10.1136/bjsports-2018-100260
KANON	Secondary analysis	Meniscal damage prevention	Snoeker BA 2020. Does early anterior cruciate ligament reconstruction prevent development of meniscal damage? Results from a secondary analysis of a randomised controlled trial. Br J Sports Med	31653779	10.1136/bjsports-2019-101125
KANON	Secondary analysis	MRI cartilage thickness (5-year change)	Wirth W 2021. Early anterior cruciate ligament reconstruction does not affect 5 year change in knee cartilage thickness: secondary analysis of a randomized clinical trial. Osteoarthritis Cartilage	33549723	10.1016/j.joca.2021.01.004
KANON	Secondary analysis	MRI ACL healing; PROMs	Filbay SR 2023. Evidence of ACL healing on MRI following ACL rupture treated with rehabilitation alone may be associated with better patient-reported outcomes: a secondary analysis from the KANON trial. Br J Sports Med	36328403	10.1136/bjsports-2022-105473

KANON	Follow-up report	PROMs; radiographic OA; long-term outcomes	Lohmander LS 2023. Treatment for Acute Anterior Cruciate Ligament Tear in Young Active Adults. NEJM Evid	38320141	10.1056/EVIDoa2200287
KANON	Secondary analysis	Alignment and subsequent OA	Nilsson H 2025. Varus alignment of the hip and knee 2 years after anterior cruciate ligament injury is associated with medial tibiofemoral osteoarthritis 3 years later. J Exp Orthop	39759097	10.1002/jeo2.70143

Note: Multiple publications from the same randomized cohort were treated as separate reports but counted as a single study (cohort) in the PRISMA study count.
